# Neoadjuvant Efficacy of Three Targeted Therapy Strategies for HER2-Positive Breast Cancer Based on the Same Chemotherapy Regimen

**DOI:** 10.3390/cancers14184508

**Published:** 2022-09-17

**Authors:** Jiujun Zhu, Dechuang Jiao, Chengzheng Wang, Zhenduo Lu, Xiuchun Chen, Lianfang Li, Xianfu Sun, Li Qin, Xuhui Guo, Chongjian Zhang, Jianghua Qiao, Min Yan, Shude Cui, Zhenzhen Liu

**Affiliations:** Department of Breast Disease, Henan Breast Cancer Center, Affiliated Cancer Hospital of Zhengzhou University & Henan Cancer Hospital, Zhengzhou 450008, China

**Keywords:** breast cancer, human epithelial growth factor receptor 2, neoadjuvant therapy, pathological complete response, pyrotinib, pertuzumab

## Abstract

**Simple Summary:**

Approximately 15–25% of breast cancers are human epidermal growth factor receptor 2 (HER2)-positive. With the progress in medicine, promising results have been shown by dual targeted therapy with new drugs in the neoadjuvant setting. In our study, we compared the effectiveness of three neoadjuvant targeted therapy strategies (H + Py, trastuzumab plus pyrotinib; H, trastuzumab; HP, trastuzumab plus pertuzumab) based on the same chemotherapy regimen (TC, docetaxel and carboplatin) for HER2-positive early breast cancer. The pathological complete response (pCR) rate was 55.6% in the TCH + Py cohort, 32.7% in the TCH cohort, and 56.6% in the TCHP cohort. The pCR rate was higher with TCH + Py than with TCH. There was no significant difference in pCR rate between the TCH + Py and TCHP cohorts.

**Abstract:**

(1) Background: The objective of our study was to provide evidence for choosing the optimal neoadjuvant therapy strategies for patients with human epidermal growth factor receptor 2 (HER2)-positive early breast cancer. Three neoadjuvant targeted therapy strategies (H + Py, trastuzumab plus pyrotinib; H, trastuzumab; HP, trastuzumab plus pertuzumab) based on the same chemotherapy regimen (TC, docetaxel and carboplatin) were included in the present study; (2) Methods: We retrospectively analyzed patients with HER2-positive breast cancer who were treated with neoadjuvant TCH + Py, TCH or TCHP, followed by surgery. The outcome was the pathological complete response (pCR) rate; (3) Results: In total, 545 patients were enrolled. The pCR rate was 55.6% (35/63) in the TCH + Py cohort, 32.7% (93/284) in the TCH cohort, and 56.6% (112/198) in the TCHP cohort. The multivariate analysis showed that patients who received TCH had less possibility to achieve pCR than those who received TCH + Py (odds ratio (OR) = 0.334, 95% confidence interval (CI): 0.181–0.619, *p* < 0.001), while patients who received TCHP had comparable possibility to those who received TCH + Py (OR = 1.043, 95%CI: 0.554–1.964, *p* = 0.896); (4) Conclusions: TCH + Py provides a better pCR rate compared with TCH, and a comparable pCR rate with TCHP among patients with HER2-positive breast cancer in the neoadjuvant setting. The present study supports a novel potential treatment option for these patients. Further studies need to be explored in the future.

## 1. Introduction

Breast cancer is one of the most commonly diagnosed malignancies. Approximately 15–25% of breast cancers are human epidermal growth factor receptor 2 (HER2)-positive subtype, which is highly aggressive with worse prognosis [1]. For patients with stage II or III breast cancer, neoadjuvant therapy has been widely accepted to reduce tumor burden for surgery and provide a greater opportunity to receive breast-conserving surgery for patients with large tumor. Relevant information of drug sensitivity can also be gained to guide subsequent treatment options and improve the prognosis of patients [2]. Pathological complete response (pCR) has been used as a surrogate endpoint in most neoadjuvant trials, and is positively associated with overall survival, especially for patients with HER2-positive and triple negative breast cancer [3].

In recent years, enormous progress of neoadjuvant therapy has been achieved in HER2-positive breast cancer, which mainly includes two aspects: (1) the use of dual-targeted regimen; (2) the combination of chemotherapeutic agents and targeted agents [4,5,6,7,8]. In terms of the dual-targeted regimen, the most commonly used combination is trastuzumab plus pertuzumab. The NeoSphere and PEONY studies have demonstrated improved pCR rate with this combination, which has become a standard of care for patients with HER2-positive breast cancer in the neoadjuvant setting [6,7]. Antibody-drug conjugate (ADC) agents are being considered as a component of dual-targeted regimen. ADC agents can combine chemotherapeutic agents with monoclonal antibody to work as targeted chemotherapy. However, the KRISTINE study showed a lower pCR rate in patients treated with trastuzumab emtansine (T-DM1) plus pertuzumab than in patients treated with systemic chemotherapy plus dual HER2-targeted blockade [8]. Therefore, further research is warranted to investigate whether systemic chemotherapy can be excluded from the neoadjuvant regimen for patients with HER2-positive breast cancer. Notably, many studies have paid attention to a dual-targeted regimen with trastuzumab plus tyrosine kinase inhibitor (TKI), such as lapatinib and neratinib [4,5,9,10,11].

Pyrotinib, an orally administered, irreversible novel TKI targeting HER1, HER2, and HER4, gained approval in China for the treatment of HER2-positive advanced or metastatic breast cancer in 2018. Pyrotinib can inhibit the formation of homologous/heterodimer and auto-phosphorylation of HER family by covalently binding with ATP at intracellular kinase regions, block the activation of downstream signaling pathways (RAS/RAF/MEK/MAPK and PI3K/AKT) and tumor cell cycle in G1 phase, and restrict tumor development [12,13]. Trastuzumab is a humanized IgG1 monoclonal antibody, which binds to the extracellular regions of HER2 and inhibits the formation of homodimer of HER2 and heterodimer of HER2-HER3, thus blocking the activation of downstream signaling pathways (such as PI3K/AKT) and restricting tumor cell growth [14]. Given that pyrotinib and trastuzumab target different epitopes of HER2 protein to inhibit its downstream signaling pathway, theoretically, they have complementary mechanisms of action to behave as HER2 antagonists, and a better antitumor activity will be seen when these two agents are combined together than either agent used alone [15]. Significant clinical benefits have been demonstrated when pyrotinib was utilized for patients with HER2-positive metastatic breast cancer in the phase III PHOEBE and PHENIX studies [16,17]. In our previous Panphila study, we found a pCR rate of 55.1% in the modified intention-to-treat population (n = 69) treated with TCH + Py (docetaxel, carboplatin, trastuzumab, and pyrotinib) [18]. The phase III PHEDRA study has also demonstrated that the dual-targeted neoadjuvant regimen with pyrotinib and trastuzumab plus chemotherapy can provide clinical benefits in patients with HER2-positive breast cancer [19].

While the overall treatment strategy and drug options are relatively well-defined for HER2-positive breast cancer, and the dual-targeted therapy with trastuzumab plus pertuzumab is still the standard neoadjuvant regimen, the development of more drugs such as TKIs can enrich clinical treatment options. It is also unclear how to choose the best partner of trastuzumab (pertuzumab or TKI). Therefore, we performed this population-based study to compare the effectiveness of three neoadjuvant targeted therapy strategies (trastuzumab plus pyrotinib, trastuzumab, or trastuzumab plus pertuzumab) based on the same chemotherapy regimen (docetaxel and carboplatin), which can provide evidence on precise neoadjuvant therapy strategies for patients with HER2-positive breast cancer.

## 2. Materials and Methods

### 2.1. Patients

Data on patients receiving neoadjuvant TCH + Py were from our previous Panphila study (NCT 03735966) [18]. The clinical records of patients with HER2-positive breast cancer who received neoadjuvant TCH (docetaxel, carboplatin, and trastuzumab) or TCHP (docetaxel, carboplatin, trastuzumab, and pertuzumab) were retrospectively reviewed at Henan Cancer Hospital between January 2014 and May 2021. All procedures involving human participants in the present study were carried out according to the Declaration of Helsinki (as revised in 2013). The approval of this study has been obtained from the ethics committee of Henan Cancer Hospital (No. 2017407).

Key inclusion criteria included: (1) female patients aged 18 to 70 years; (2) Eastern Cooperative Oncology Group (ECOG) performance status of 0–1; (3) confirmed positive HER2 status by immunohistochemistry (IHC) or fluorescence in situ hybridization (FISH); (4) early-stage or locally advanced breast cancer; (5) known hormone receptor (HR) status; (6) known Ki-67 level, categorized as low (≤30%) and high (>30%) expression based on the ratio of positive cells to all tumor cells in 10 high-power fields [20,21]; (7) normal hematologic, hepatic, and renal function; (8) neoadjuvant treatment using the TCH + Py, TCH or TCHP for at least 4 cycles. The exclusion criteria were: (1) bilateral breast cancer; (2) inflammatory breast cancer; (3) pregnancy; (4) primary breast tumor or axillary lymph nodes resection before receiving neoadjuvant therapy. 

Patients in the TCH cohort received docetaxel 75 mg/m^2^ on day 1, carboplatin 6 mg/mL/min on day 1, and trastuzumab 8 mg/kg loading dose followed by 6 mg/kg maintenance dose on day 1 of each 21-day cycle. Patients in the TCH + Py cohort received pyrotinib 400 mg once a day plus the same TCH regimen. In the TCHP cohort, patients received pertuzumab 840 mg loading dose followed by 420 mg maintenance dose on day 1 plus the same TCH regimen. All drugs were administered intravenously except that pyrotinib was given orally. All treatments were given for at least 4 cycles. Surgery was performed 2 weeks after neoadjuvant therapy.

The outcome was pCR rate, and pCR was defined as the absence of residual invasive tumor cells in the specimen from the breast and axillary lymph nodes (ypT0/is ypN0) [3]. Each specimen was stained with hematoxylin and eosin (HE) after neoadjuvant therapy and surgery. 

### 2.2. Statistical Analysis

Categorical variables were expressed as frequency and percentage, and compared among the three cohorts using the Chi-square test. To determine the factors associated with pCR, the univariate analysis using the Chi-square test and multivariate analysis using the logistic regression model were performed in the total population and exploratory subgroups by HR status and HER2 status, respectively. Two-sided *p* < 0.05 was considered statistically significant. SPSS 23.0 (IBM, Armonk, NY, USA) was used for all the statistical analyses.

## 3. Results

### 3.1. Patients’ Characteristics

**Figure 1:**
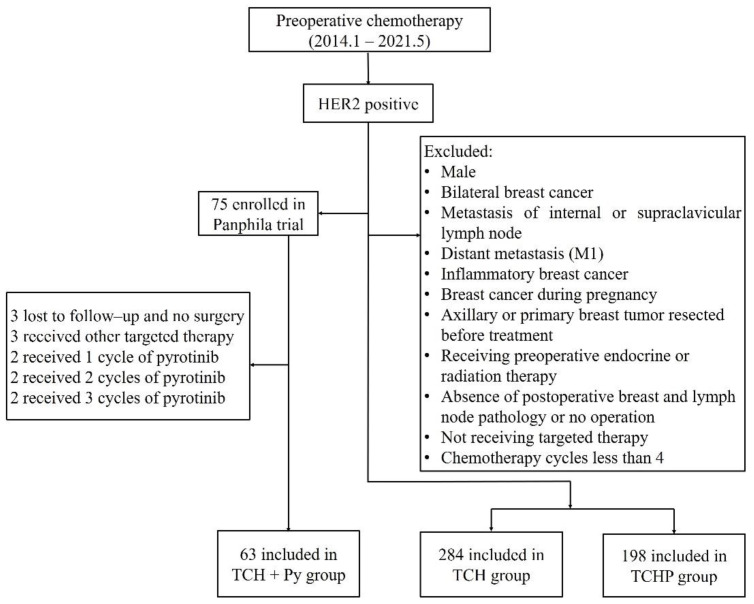
Patient flow diagram.

In total, 545 patients were included in our study between January 2014 and May 2021, of which 63 were in the TCH + Py cohort, 284 in the TCH cohort, and 198 in the TCHP cohort (Figure 1). Of 545 patients, 302 (55.4%) were below 50 years of age, 315 (57.8%) were premenopausal, and most had T2 (76.1%) tumor and N1 (45.5%) lymph node status; 334 (61.3%) patients had HR-positive breast cancer and 108 (19.8%) patients had HER2 IHC 2+ disease with confirmed amplification by FISH. The baseline characteristics are shown in Table 1.

### 3.2. Comparison of Three Targeted Therapy Strategies by Univariate Analysis

Univariate analysis revealed statistically significant difference in pCR rate between the three treatment cohorts (*p* < 0.001). Patients who received TCH + Py had a higher pCR rate than those who received TCH (55.6% vs. 32.7%), and had a comparable pCR rate with those who received TCHP (55.6% vs. 56.6%) (Table 2). Furthermore, earlier T stage (*p* = 0.023), earlier N stage (*p* = 0.036), HR-negative status (*p* < 0.001), HER2 IHC 3+ (*p* < 0.001), and high Ki-67 expression (*p* = 0.015) were potentially associated with a higher pCR rate. 

### 3.3. Comparison of Three Targeted Therapy Strategies by Multivariate Analysis 

Significant difference in pCR rate among the three cohorts (*p* < 0.001) persisted after using multivariate analysis to adjust confounding factors, including T stage, N stage, HR status, HER2 status, and Ki-67 expression (Table 3). Patients who received TCH had less possibility to achieve pCR than those who received TCH + Py (odds ratio (OR) = 0.334, 95%CI: 0.181–0.619, *p* < 0.001), while patients who received TCHP had comparable possibility to those who received TCH + Py (OR = 1.043, 95%CI: 0.554–1.964, *p* = 0.896) (Table 3). The possibility to achieve pCR in patients with HR-negative breast cancer was more than 2 times higher than that in patients with HR-positive breast cancer (OR = 2.033, 95%CI: 1.377–2.994, *p* < 0.001). Moreover, 4.7 times higher possibility to achieve pCR was observed in patients with HER2 IHC 3+ breast cancer than in patients with HER2 IHC 2+ breast cancer (OR = 4.726, 95%CI: 2.706–8.253, *p* < 0.001). 

### 3.4. Subgroup Analysis

The exploratory analyses of pCR rate were performed in subgroups by HR status and HER2 status, respectively. The pCR rate was 85.0% with TCH + Py, 38.7% with TCH, and 71.3% with TCHP in patients with HR-negative breast cancer, with statistical difference among the three targeted therapy strategies (*p* < 0.001). For patients with HR-positive breast cancer, the pCR rate was 41.9% with TCH + Py, 28.9% with TCH, and 46.6% with TCHP, also with statistical difference among the three targeted therapy strategies (*p* = 0.007) (Table S1). The multivariate analysis demonstrated that the TCH cohort had less possibility to achieve pCR than the TCH + Py cohort for patients with HR-negative breast cancer (OR = 0.108, 95%CI: 0.027–0.424, *p* = 0.001). However, comparable possibility was observed between the TCH + Py cohort and TCH cohort in patients with HR-positive breast cancer (OR = 0.527, 95%CI: 0.247–1.124, *p* = 0.097). Patients who received TCHP had comparable possibility to achieve pCR with patients who received TCH + Py in both HR-negative (OR = 0.469, 95%CI: 0.115–1.908, *p* = 0.290) and HR-positive (OR = 1.337, 95%CI: 0.616–2.900, *p* = 0.462) subgroups (Table 4).

For patients with HER2 IHC 2+ disease, the pCR rate was 33.3% with TCH + Py, 7.5% with TCH, and 27.9% with TCHP, with statistical difference among the three targeted therapy strategies (*p* = 0.024). For patients with HER2 IHC 3+ disease, the pCR rate was 60.8% with TCH + Py, 38.5% with TCH, and 64.5% with TCHP, also with statistical difference among the three targeted therapy strategies (*p* < 0.001) (Table S2). The multivariate analysis demonstrated that the TCH cohort had less possibility to achieve pCR than the TCH + Py cohort for patients with HER2 IHC 3+ disease (OR = 0.371, 95%CI: 0.192–0.716, *p* = 0.003), but comparable possibility was observed between these two cohorts in patients with HER2 IHC 2+ disease (OR = 0.188, 95%CI: 0.031–1.137, *p* = 0.069). Patients who received TCHP had comparable possibility to achieve pCR with patients who received TCH + Py in both HER2 IHC 2+ (OR = 0.764, 95%CI: 0.144–4.061, *p* = 0.752) and HER2 IHC 3+ (OR = 1.096, 95%CI: 0.552–2.173, *p* = 0.794) subgroups (Table 5).

## 4. Discussion

Over the past decades, HER2-targeted therapy has significantly improved the outcomes of breast cancer, and its efficacy has been demonstrated in several clinical studies [4,5,9,10,11]. Furthermore, the PHEDRA study firstly confirmed the efficacy of neoadjuvant pyrotinib in patients with HER2-positive breast cancer with a randomized phase 3 design [19]. Despite that dual-targeted therapy with trastuzumab and pertuzumab remains the standard neoadjuvant regimen for HER2-positive breast cancer, the use of pyrotinib can enrich clinical treatment options. However, evidence on precise targeted therapy strategies for HER2-positive breast cancer is still lacking. To address this problem, we carried out this study to compare the effectiveness of TCH + Py, TCH, and TCHP regimens for the neoadjuvant treatment of HER2-positive breast cancer, and the results showed a pCR rate of 55.6%, 32.7%, and 56.6%, respectively. Multivariate analyses showed that patients who received TCH + Py had a greater opportunity to achieve pCR than patients who received TCH in the total population. Similar results were observed in the HR-negative and HER2 IHC 3+ subgroups, respectively. However, only a trend favoring TCH + Py without statistical significance was found in the HR-positive or HER2 IHC 2+ subgroup. Comparable possibility to achieve pCR was observed between the TCH + Py and TCHP cohorts, whether in the total population or in subgroups by HR status or HER2 status.

Neoadjuvant trastuzumab combined with chemotherapy has greatly increased the pCR rate in patients with HER2-positive breast cancer [22,23]. Our results revealed a higher pCR rate in patients treated with TCH + Py than patients who received TCH, especially in HR-negative or HER2 IHC 3+ patients. These results were consistent with previous clinical trials [4,6,24]. Meanwhile, only an increasing trend of pCR rate was found in HR-positive patients, with no statistical difference. Given that the clinical benefit of neoadjuvant TKI therapy is independent of HR status, as demonstrated by previous study [25], the results shown in our study might be attributed to the small sample size in the TCH + Py cohort and lack of statistical power. On one hand, the present study showed that dual HER2-blockade with pyrotinib and trastuzumab was highly active. It also suggests that in comparison with trastuzumab alone, the combination of two targeted agents, including pyrotinib, was associated with a higher pCR rate, as supported by previous studies [4,6,18]. On the other hand, a high pCR rate up to 71.3% (TCHP) or 85.0% (TCH + Py) was observed in patients with HR-negative breast cancer who were treated with chemotherapy plus dual HER2-blockade. Similar results have been reported in the TRYPHAENA and KRISTINE studies [8,26]. Results from previous and the present studies reflect that patients with HR-negative, HER2-positive breast cancer are more likely to achieve pCR with chemotherapy plus dual HER2-blockade than those with HR-positive, HER2-positive breast cancer. Thus, de-escalation treatment might be more feasible in patients with HR-positive disease than in those with HR-negative disease [27,28].

A network meta-analysis including 10 neoadjuvant studies showed that TKI did not result in statistically different pCR rate compared to pertuzumab when combined with trastuzumab and chemotherapy [29]. As expected, we found a comparable pCR rate between patients who received TCH + Py and those treated with TCHP, regardless of the HR status or HER2 status. The pCR rate with neoadjuvant TCHP in our study was 56.6%, which was consistent with the results from the KRISTINE trial (55.7%) and a retrospective study (55.6%) [8,30], but higher than that in the PEONY (39.3%) and NeoSphere (breast pCR 45.8%) studies [6,7]. It should be noted that only 4 cycles of pertuzumab, trastuzumab, and docetaxel were performed in patients from these studies. In contrast, a higher pCR rate of 67% was observed in the TRAIN-2 study which enrolled patients to receive up to 9 cycles of neoadjuvant therapy [31]. In addition, the effect of carboplatin as a component of chemotherapy regimen cannot be ruled out [32]. Therefore, in line with previous studies, our study suggests the similar effectiveness of TCH + Py compared with TCHP in the neoadjuvant treatment of HER2-positive breast cancer. Although neoadjuvant pertuzumab has been used as the standard of care for patients with locally advanced HER2-positive breast cancer, our study suggests that pyrotinib can be a novel option for patients who cannot use pertuzumab possibly due to low tolerance or high price [33]. Nevertheless, more studies are needed to validate our speculation in the future.

Notably, many studies have demonstrated that dual-targeted therapy with trastuzumab plus TKI or pertuzumab can lead more patients to reach pCR [4,5]. A meta-analysis including 9 studies confirmed that the pCR rate of dual HER2-blockade with trastuzumab plus TKI (lapatinib or neratinib) or pertuzumab was higher than that of trastuzumab single-targeted therapy (risk ratio = 1.31, 95%CI: 1.21–1.43, *p* < 0.001) [25]. However, the efficacy between TKI plus trastuzumab and pertuzumab plus trastuzumab has only been compared in limited studies, without confirmative conclusions. The TEAL study only enrolled 30 evaluable patients, which reported that the combination of T-DM1, lapatinib, and nab-paclitaxel was more effective than trastuzumab, pertuzumab, and paclitaxel as neoadjuvant therapy in HER2-positive breast cancer, especially for estrogen receptor-positive patients [34]. Another preclinical study using HER2-positive breast cancer xenograft models showed that neratinib plus trastuzumab had a better antitumor effect than pertuzumab plus trastuzumab [35]. Importantly, the efficacy of TKI was observed in patients with pertuzumab resistance in the NALA study [36]. To our knowledge, our study is the first one to compare the neoadjuvant effectiveness between pyrotinib and pertuzumab based on the same backbone regimen. As pyrotinib is a small-molecule TKI and pertuzumab is a macromolecular antibody which prevents HER2 receptor dimerization, our study supports an alternative treatment option for patients with HER2-positive breast cancer. In addition, the adoption of pyrotinib after resistance to neoadjuvant chemotherapy and pertuzumab needs to be further investigated.

With increasing evidence on the efficacy of TKI in breast cancer, identifying individual response is crucial. How to precisely identify patients who are sensitive to pertuzumab or TKI is one of the issues that needs to be addressed. In the Panphila trial, we found that higher baseline infiltration of stromal immune cells was significantly associated with the neoadjuvant efficacy of pyrotinib [18]. The exploratory analysis from the NeoALTTO study indicates that lymphocyte-specific kinase or transcriptional similarity of biological pathways can predict pCR to neoadjuvant lapatinib and trastuzumab [37,38]. In addition, higher pCR rate with pertuzumab was observed in patients with low expression of some immune markers at baseline, such as MHC1 and CTLA4 [39]. These studies suggest that patient’s tumor microenvironment may influence the sensitivity to neoadjuvant therapy, which needs further validation in future studies. Markers that can more effectively predict the sensitivity to different targeted therapies also need to be developed.

The main strength of the present study is that we conducted a large study to compare the neoadjuvant effectiveness of three targeted therapy strategies (trastuzumab plus pyrotinib, trastuzumab, or trastuzumab plus pertuzumab) based on the same chemotherapy regimen in patients with HER2-positive breast cancer. However, several limitations in this study should be noted. Firstly, our study was conducted retrospectively and the sample size was relatively small in the TCH + Py cohort. Secondly, we did not collect data on adverse events, thus the safety profiles among these three treatment cohorts have not been compared. Previous studies have reported that the most common grade 3 to 4 adverse events were diarrhea, anemia, and vomiting in patients who received TCH + Py, and neutropenia, febrile neutropenia, and anemia in patients who received TCHP [8,18]. A meta-analysis indicated that the incidence of serious adverse events following dual-targeted therapy was comparable to that following trastuzumab single-targeted therapy, which supports the use of dual HER2-blockade [25]. Finally, we did not analyze survival data, which will be reported in our future studies.

## 5. Conclusions

In summary, TCH + Py provides a higher pCR rate compared with TCH, and a comparable pCR rate with TCHP for patients with HER2-positive breast cancer in the neoadjuvant setting. The present study supports a novel potential treatment option for these patients. Further studies comparing pyrotinib with pertuzumab are warranted in the future. In addition, biomarkers predicting patient susceptibility to different targeted therapy regimens also need to be explored.

## Figures and Tables

**Table 1:** Characteristics of patients in three cohorts.

Characteristics		Total (N = 545)	TCH + Py (N = 63)	TCH (N = 284)	TCHP (N = 198)	*p*
Age (years)	<50	302 (55.4)	35 (55.6)	161 (56.7)	106 (53.5)	0.790
	≥50	243 (44.6)	28 (44.4)	123 (43.3)	92 (46.5)	
Menopausal status	Premenopausal	315 (57.8)	36 (57.1)	167 (58.8)	112 (56.6)	0.882
	Postmenopausal	230 (42.2)	27 (42.9)	117 (41.2)	86 (43.4)	
T	T1	38 (7.0)	0	26 (9.2)	12 (6.1)	0.001
	T2	415 (76.1)	58 (92.1)	204 (71.8)	153 (77.3)	
	T3	64 (11.7)	5 (7.9)	37 (13.0)	22 (11.1)	
	T4	28 (5.1)	0	17 (6.0)	11 (5.6)	
N	N0	115 (21.1)	18 (28.6)	54 (19.0)	43 (21.7)	0.003
	N1	248 (45.5)	34 (54)	135 (47.5)	79 (39.9)	
	N2	77 (14.1)	9 (14.3)	44 (15.5)	24 (12.1)	
	N3	105 (19.3)	2 (3.2)	51 (18.0)	52 (26.3)	
HR status	Negative	211 (38.7)	20 (31.7)	111 (39.1)	80 (40.4)	0.462
	Positive	334 (61.3)	43 (68.3)	173 (60.9)	118 (59.6)	
HER2 status	IHC 2+	108 (19.8)	12 (19.0)	53 (18.7)	43 (21.7)	0.701
	IHC 3+	437 (80.2)	51 (81.0)	231 (81.3)	155 (78.3)	
Ki-67	Low expression	117 (21.5)	13 (20.6)	63 (22.2)	41 (20.7)	0.914
	High expression	428 (78.5)	50 (79.4)	221 (77.8)	157 (79.3)	

TCH + Py, docetaxel, carboplatin, trastuzumab, and pyrotinib; TCH, docetaxel, carboplatin, and trastuzumab; TCHP, docetaxel, carboplatin, trastuzumab, and pertuzumab; T, tumor; N, node; HR, hormone receptor; HER2, human epidermal growth factor receptor 2; IHC, immunohistochemistry.

**Table 2:** Univariate analyses of factors associated with pCR.

Characteristics		N	Non-pCR	pCR	*p*
Age (years)	<50	302	179 (59.3)	123 (40.7)	0.083
	≥50	243	126 (51.9)	117 (48.1)	
Menopausal status	Premenopausal	315	185 (58.7)	130 (41.3)	0.128
	Postmenopausal	230	120 (52.2)	110 (47.8)	
T	T1	38	17 (44.7)	21 (55.3)	0.023
	T2	415	224 (54.0)	191 (46.0)	
	T3	64	45 (70.3)	19 (29.7)	
	T4	28	19 (67.9)	9 (32.1)	
N	N0	115	52 (45.2)	63 (54.8)	0.036
	N1	248	145 (58.5)	103 (41.5)	
	N2	77	50 (64.9)	27 (35.1)	
	N3	105	58 (55.2)	47 (44.8)	
HR status	Negative	211	94 (44.5)	117 (55.5)	<0.001
	Positive	334	211 (63.2)	123 (36.8)	
HER2 status	IHC 2+	108	88 (81.5)	20 (18.5)	<0.001
	IHC 3+	437	217 (49.7)	220 (50.3)	
Ki-67	Low expression	117	77 (65.8)	40 (34.2)	0.015
	High expression	428	228 (53.3)	200 (46.7)	
Regimen	TCH + Py	63	28 (44.4)	35 (55.6)	<0.001
	TCH	284	191 (67.3)	93 (32.7)	
	TCHP	198	86 (43.4)	112 (56.6)	
Total		545	305 (56.0)	240 (44.0)	

pCR, pathological complete response; T, tumor; N, node; HR, hormone receptor; HER2, human epidermal growth factor receptor 2; IHC, immunohistochemistry; TCH + Py, docetaxel, carboplatin, trastuzumab, and pyrotinib; TCH, docetaxel, carboplatin, and trastuzumab; TCHP, docetaxel, carboplatin, trastuzumab, and pertuzumab.

**Table 3:** Multivariate analysis of factors associated with pCR.

Variables		OR	95%CI	*p*
T	T1	1		0.018
	T2	0.537	0.252–1.144	0.107
	T3	0.252	0.100–0.631	0.003
	T4	0.365	0.120–1.116	0.077
N	N0	1		0.055
	N1	0.557	0.340–0.913	0.020
	N2	0.442	0.231–0.847	0.014
	N3	0.639	0.351–1.162	0.142
HR status	Positive	1	1.377–2.994	
	Negative	2.033	1.377–2.994	<0.001
HER2 status	IHC 2+	1		
	IHC 3+	4.726	2.706–8.253	<0.001
Ki-67	Low expression	1		
	High expression	1.670	1.043–2.673	0.033
Regimen	TCH + Py	1		<0.001
	TCH	0.334	0.181–0.619	<0.001
	TCHP	1.043	0.554–1.964	0.896

pCR, pathological complete response; OR, odds ratio; CI, confidence interval; T, tumor; N, node; HR, hormone receptor; HER2, human epidermal growth factor receptor 2; IHC, immunohistochemistry; TCH + Py, docetaxel, carboplatin, trastuzumab, and pyrotinib; TCH, docetaxel, carboplatin, and trastuzumab; TCHP, docetaxel, carboplatin, trastuzumab, and pertuzumab.

**Table 4:** Multivariate analyses of factors associated with pCR in subgroup by HR status.

Variables		HR Negative			HR Positive		
		OR	95%CI	*p*	OR	95%CI	*p*
T	T1	1		0.297	1		0.066
	T2	0.860	0.286–2.588	0.788	0.372	0.132–1.049	0.061
	T3	0.385	0.101–1.467	0.162	0.175	0.048–0.638	0.008
	T4	0.442	0.069–2.838	0.389	0.297	0.071–1.246	0.097
N	N0	1		0.706	1		0.098
	N1	0.653	0.275–1.550	0.334	0.515	0.278–0.955	0.035
	N2	0.571	0.184–1.774	0.333	0.413	0.184–0.927	0.032
	N3	0.592	0.217–1.62	0.307	0.695	0.326–1.481	0.345
HER2 status	IHC 2+	1			1		
	IHC 3+	3.577	1.296–9.872	0.014	5.689	2.811–11.512	<0.001
Ki-67	Low expression	1			1		
	High expression	2.722	1.204–6.152	0.016	1.240	0.692–2.223	0.470
Regimen	TCH + Py	1		<0.001	1		0.003
	TCH	0.108	0.027–0.424	0.001	0.527	0.247–1.124	0.097
	TCHP	0.469	0.115–1.908	0.290	1.337	0.616–2.900	0.462

pCR, pathological complete response; HR, hormone receptor; OR, odds ratio; CI, confidence interval; T, tumor; N, node; HER2, human epidermal growth factor receptor 2; IHC, immunohistochemistry; TCH + Py, docetaxel, carboplatin, trastuzumab and pyrotinib; TCH, docetaxel, carboplatin, and trastuzumab; TCHP, docetaxel, carboplatin, trastuzumab, and pertuzumab.

**Table 5:** Multivariate analyses of factors associated with pCR in subgroup by HER2 status.

Variables		HER2 IHC 2+			HER2 IHC 3+		
		OR	95%CI	*p*	OR	95%CI	*p*
T	T1	1		0.606	1		0.042
	T2	0.311	0.035–2.750	0.293	0.592	0.266–1.320	0.200
	T3	0.151	0.010–2.329	0.176	0.273	0.103–0.723	0.009
	T4	0.000		0.999	0.429	0.134–1.379	0.155
N	N0	1		0.130	1		0.200
	N1	0.283	0.070–1.148	0.077	0.638	0.377–1.081	0.095
	N2	0.212	0.028–1.603	0.133	0.509	0.256–1.013	0.054
	N3	0.178	0.034–0.919	0.039	0.797	0.416–1.527	0.494
HR status	Negative	1			1		
	Positive	0.273	0.081–0.918	0.036	0.539	0.357–0.815	0.003
Ki-67	Low expression	1			1		
	High expression	1.687	0.414–6.873	0.465	1.691	1.023–2.797	0.041
Regimen	TCH + Py	1		0.077	1		<0.001
	TCH	0.188	0.031–1.137	0.069	0.371	0.192–0.716	0.003
	TCHP	0.764	0.144–4.061	0.752	1.096	0.552–2.173	0.794

pCR, pathological complete response; HER2, human epidermal growth factor receptor 2; IHC, immunohistochemistry; OR, odds ratio; CI, confidence interval; T, tumor; N, node; HR, hormone receptor; TCH + Py, docetaxel, carboplatin, trastuzumab, and pyrotinib; TCH, docetaxel, carboplatin, and trastuzumab; TCHP, docetaxel, carboplatin, trastuzumab, and pertuzumab.

## Data Availability

The data presented in this study are available on request from the corresponding author. The data are not publicly available due to ongoing studies and for patient privacy.

## Supplementary Materials

The following supporting information can be downloaded at: https://www.mdpi.com/article/10.3390/cancers14184508/s1, Table S1: Univariate analyses of factors associated with pCR in subgroup by HR status, Table S2: Univariate analyses of factors associated with pCR in subgroup by HER2 status.
