# Supplementary material for: Neoadjuvant Efficacy of Three Targeted Therapy Strategies for HER2-Positive Breast Cancer Based on the Same Chemotherapy Regimen
Source: Cancers (Basel). 2022 Sep 17;14(18):4508. doi: 10.3390/cancers14184508 (PMC9497101; doi:10.3390/cancers14184508)
Supplement: Supplementary file 1 [file cancers-14-04508-s001.zip › cancers-1844629-supplementary.pdf]

Table S1 Univariate analyses of factors associated with pCR in subgroup by HR status

Variables		HR negative				HR positive			
		N	Non-pCR	pCR	P	N	Non-pCR	pCR	P
Age (years)	<50	104	53(51.0)	51(49.0)	0.065	198	126(63.6)	72(36.4)	0.832
	≥50	107	41(38.3)	66(61.7)		136	85(62.5)	51(37.5)	
Menopausal status	Premenopausal	115	55(47.8)	60(52.2)	0.295	200	130(65.0)	70(35.0)	0.398
	Postmenopausal	96	39(40.6)	57(59.4)		134	81(60.4)	53(39.6)	
T	T1	19	8(42.1)	11(57.9)	0.098	19	9(47.4)	10(52.6)	0.161
	T2	154	62(40.3)	92(59.7)		261	162(62.1)	99(37.9)	
	T3	29	18(62.1)	11(37.9)		35	27(77.1)	8(22.9)	
	T4	9	6(66.7)	3(33.3)		19	13(68.4)	6(31.6)	
N	N0	40	14(35.0)	26(65.0)	0.469	75	38(50.7)	37(49.3)	0.051
	N1	101	45(44.6)	56(55.4)		147	100(68.0)	47(32.0)	
	N2	26	14(53.8)	12(46.2)		51	36(70.6)	15(29.4)	
	N3	44	21(47.7)	23(52.3)		61	37(60.7)	24(39.3)	
HER2 status	IHC 2+	25	16(64.0)	9(36.0)	0.042	83	72(86.7)	11(13.3)	<0.001
	IHC 3+	186	78(41.9)	108(58.1)		251	139(55.4)	112(44.6)	
Ki-67	Low expression	40	26(65.0)	14(35.0)	0.005	77	51(66.2)	26(33.8)	0.526
	High expression	171	68(39.8)	103(60.2)		257	160(62.3)	97(37.7)	
Regimen	TCH + Py	20	3(15.0)	17(85.0)	<0.001	43	25(58.1)	18(41.9)	0.007
	TCH	111	68(61.3)	43(38.7)		173	123(71.1)	50(28.9)	
	TCHP	80	23(28.8)	57(71.3)		118	63(53.4)	55(46.6)	

pCR, pathological complete response; HR, hormone receptor; T, tumor; N, node; HER2, human epidermal growth factor receptor 2; IHC, immunohistochemistry; TCH + Py, docetaxel, carboplatin, trastuzumab and pyrotinib; TCH, docetaxel, carboplatin and trastuzumab; TCHP, docetaxel, carboplatin, trastuzumab and pertuzumab.

Table S2 Univariate analyses of factors associated with pCR in subgroup by HER2 status

Variables		HER2 IHC 2+				HER2 IHC 3+			
		N	Non-pCR	pCR	P	N	Non-pCR	pCR	P
Age (years)	<50	58	48(82.8)	10(17.2)	0.713	244	131(53.7)	113(46.3)	0.058
	≥50	50	40(80.0)	10(20.0)		193	86(44.6)	107(55.4)	
Menopausal status	Premenopausal	59	48(81.4)	11(18.6)	0.971	256	137(53.5)	119(46.5)	0.055
	Postmenopausal	49	40(81.6)	9(18.4)		181	80(44.2)	101(55.8)	
T	T1	6	4(66.7)	2(33.3)	0.550	32	13(40.6)	19(59.4)	0.034
	T2	84	68(81.0)	16(19.0)		331	156(47.1)	175(52.9)	
	T3	13	11(84.6)	2(15.4)		51	34(66.7)	17(33.3)	
	T4	5	5(100)	0		23	14(60.9)	9(39.1)	
N	N0	20	12(60.0)	8(40.0)	0.076	95	40(42.1)	55(57.9)	0.125
	N1	48	41(85.4)	7(14.6)		200	104(52.0)	96(48.0)	
	N2	15	13(86.7)	2(13.3)		62	37(59.7)	25(40.3)	
	N3	25	22(88.0)	3(12.0)		80	36(45.0)	44(55.0)	
HR status	Negative	25	16(64.0)	9(36.0)	0.014	186	78(41.9)	108(58.1)	0.006
	Positive	83	72(86.7)	11(13.3)		251	139(55.4)	112(44.6)	
Ki-67	Low expression	27	23(85.2)	4(14.8)	0.569	90	54(60.0)	36(40.0)	0.029
	High expression	81	65(80.2)	16(19.8)		347	163(47.0)	184(53.0)	
Regimen	TCH + Py	12	8(66.7)	4(33.3)	0.024	51	20(39.2)	31(60.8)	<0.001
	TCH	53	49(92.5)	4(7.5)		231	142(61.5)	89(38.5)	
	TCHP	43	31(72.1)	12(27.9)		155	55(35.5)	100(64.5)	

pCR, pathological complete response; HER2, human epidermal growth factor receptor 2; IHC, immunohistochemistry; T, tumor; N, node; HR, hormone receptor; TCH + Py, docetaxel, carboplatin, trastuzumab and pyrotinib; TCH, docetaxel, carboplatin and trastuzumab; TCHP, docetaxel, carboplatin, trastuzumab and pertuzumab.
